# In Vitro Antiviral Activity of α-Mangostin against Dengue Virus Serotype-2 (DENV-2)

**DOI:** 10.3390/molecules26103016

**Published:** 2021-05-19

**Authors:** Kingshuk Panda, Kalichamy Alagarasu, Poonam Patil, Megha Agrawal, Ashwini More, Naveen V. Kumar, Prathama S. Mainkar, Deepti Parashar, Sarah Cherian

**Affiliations:** 1 ICMR-National Institute of Virology, 20-A, Dr. Ambedkar Road, Pune 411001, Maharashtra, India; kingshukpanda7@gmail.com (K.P.); alagarasu@gmail.com (K.A.); poonamshewale07@rediffmail.com (P.P.); meghabi@gmail.com (M.A.); ashwini05.s@gmail.com (A.M.); 2 CSIR-Indian Institute of Chemical Technology (CSIR-IICT), Hyderabad 500007, Telangana, India; vnkumar78@iict.res.in (N.V.K.); prathama.iict@gov.in (P.S.M.)

**Keywords:** antiviral, α-Mangostin, dengue, xanthonoids, FFU assay, quantitative RT-PCR, IFA

## Abstract

Dengue virus (DENV), a member of the family Flaviviridae, is a threat for global health as it infects more than 100 million people yearly. Approved antiviral therapies or vaccines for the treatment or prevention of DENV infections are not available. In the present study, natural compounds were screened for their antiviral activity against DENV by in vitro cell line-based assay. α-Mangostin, a xanthanoid, was observed to exert antiviral activity against DENV-2 under pre-, co- and post-treatment testing conditions. The antiviral activity was determined by foci forming unit (FFU) assay, quantitative RT-PCR and cell-based immunofluorescence assay (IFA). A complete inhibition of DENV-2 was observed at 8 µM under the co-treatment condition. The possible inhibitory mechanism of α-Mangostin was also determined by docking studies. The molecular docking experiments indicate that α-Mangostin can interact with multiple DENV protein targets such as the NS5 methyltransferase, NS2B-NS3 protease and the glycoprotein E. The in vitro and in silico findings suggest that α-Mangostin possesses the ability to suppress DENV-2 production at different stages of its replication cycle and might act as a prophylactic/therapeutic agent against DENV-2.

## 1. Introduction

Dengue is the most wide-spread and fastest growing vector-borne viral infection in tropical and subtropical regions. The World Health Organization (WHO) estimates an annual incidence of approximately 100 million infections, with approximately 500,000 severe dengue cases leading to almost 25,000 deaths annually [1]. Dengue virus (DENV) with four antigenically distinct serotypes belongs to the Flaviviridae family and contains a positive-sense single-stranded RNA genome with a size of approximately 11 kb [2]. Transmission of DENV infection occurs through the bite of female mosquito vectors *Aedes aegypti* and *Aedes albopictus* [3]. Similar to other flaviviruses, the DENV RNA is released into the cytoplasm to function as a mature mRNA during infection. The encoded polyprotein is finally converted into three structural proteins (core protein, membrane-associated protein, envelope protein) and seven non-structural proteins (NS1, NS2a, NS2b, NS3, NS4a, NS4b, NS5) by both host and virus proteases [4,5].

Currently, there is no effective vaccine or drug available for the prevention and treatment of DENV infection. Supportive care with analgesics, fluid replacement and bed rest are generally prescribed for dengue patient management [6]. Several drugs such as chloroquine, celgosivir, lovastatin, balapiravir and prednisolone have been evaluated for their antiviral potential against dengue but failed to meet efficacy endpoint [7]. More than 80% of the world population depends on traditional medicine due to economic and geographical constraints [8,9]. The use of natural products has been described as traditional medicines, remedies, potions, and oils throughout history. Purified natural compounds provide a rich source for antiviral drug development. Natural compounds continue to provide unique structural diversity, increasing the opportunity to find novel low molecular weight compounds [10]. Flavonoid and xanthonoid compounds are recognized as a secondary metabolite produced by plants and already been investigated for potent biological activities such as antioxidant, anti-inflammatory, anticancer, antibacterial, antifungal and antiviral activity [11,12,13,14]. Several flavonoids have already been screened for anti-DENV property and identified as inhibitors of DENV based on in vitro studies [15,16,17,18]. The current study investigated the anti-DENV-2 activity of 47 synthesized natural compounds obtained from the National MolBank compound repository of the Council of Industrial and Scientific Research-Indian Institute of Chemical Technology (CSIR-IICT), Hyderabad. Based on in vitro screening, six compounds showed significant antiviral activity. Of these, one compound α-Mangostin, demonstrated significant anti-DENV-2 activity and was further investigated by in silico docking studies to identify the possible mechanism of action. This compound was thus identified as a potent inhibitor of DENV-2.

## 2. Results

### 2.1. Identification of Compound with Maximum Antiviral Activity

The effect of the compounds (*n* = 47) on Vero CCL81 viability was tested using MTT assay and it was found that a total of 43 compounds showed cell viability ≥80% based on the CC50 values. Based on the effect of these compounds on Vero cell viability, a 10 µM concentration was used for antiviral screening. The compounds were tested for their anti-viral activity under three conditions: pre-treatment of cells before infection, co-treatment of the virus and the cells during infection and post-treatment of cells after infection. In the culture supernatants, viral genomic RNA quantification assay was carried out to evaluate the antiviral activity of the compounds. A total of six compounds showed antiviral activity (≥1 log10 reduction) against DENV-2 under different conditions. α-Mangostin was the only compound which showed maximum reduction of viral RNA copy number under all the conditions. This compound was therefore selected for further in vitro investigation in a dose dependent manner and also analyzed for its target specificity through in silico studies.

### 2.2. Inhibitory Effect of α-Mangostin on DENV-2 Replication

**Figure 1:**
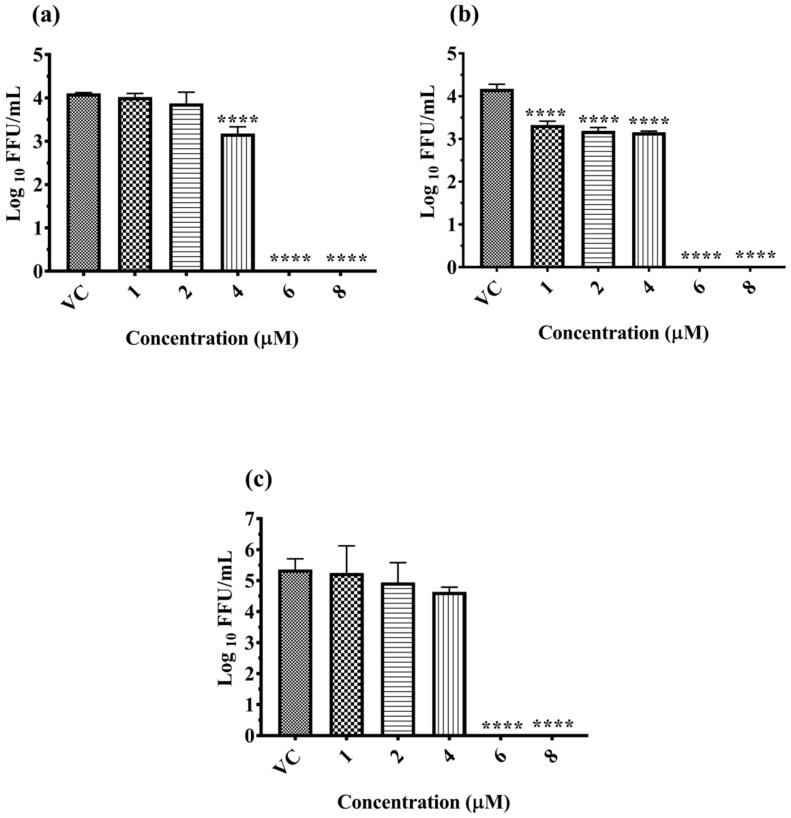
Effect of α-Mangostin on DENV-2 by focus forming unit assay under pre-treatment (**a**) co-treatment (**b**) and post-treatment (**c**) conditions. All the values are expressed as mean ± SD of three experiments. **** *p* < 0.0001, vs. control.

Dose dependent antiviral effect of α-Mangostin on DENV-2 was investigated under the following conditions: pre-treatment of cells, co-treatment of cells and the virus and post-treatment after infection. The culture supernatants were assessed for copy number of viral genomic RNA and titer of infectious virus particles to understand the effect of α-Mangostin on DENV-2 replication (Figure 1). The pre-, co- and post-treatment of cells with 6 µM and 8 µM of α-Mangostin showed a 100% reduction (*p* < 0.0001) of the virus titer compared to virus control (Figure 1a–c). A significant one log reduction in virus foci (4.10 to 3.17 mean log10 FFU/mL value) was observed in cells pre-treated with 4 µM α-Mangostin (*p* < 0.0001). A significant reduction of viral titer from 4.16 (VC) to 3.32, 3.18 and 3.15 mean log10 FFU/mL at 1 µM, 2 µM and 4 µM, respectively, was observed in the case of co-treatment (*p* < 0.0001) (Figure 1b). When the cells were treated with α-Mangostin 24 h after infection, 100% reduction of viral foci (*p* < 0.0001) was observed at 6 µM, and 8 µM. Viral foci reduced from 5.36 (VC) to 5.24, 4.94 and 4.63 mean log10 FFU/mL values at 1 µM, 2 µM and 4 µM, respectively, but the reduction was not statistically significant (Figure 1c).

**Figure 2:**
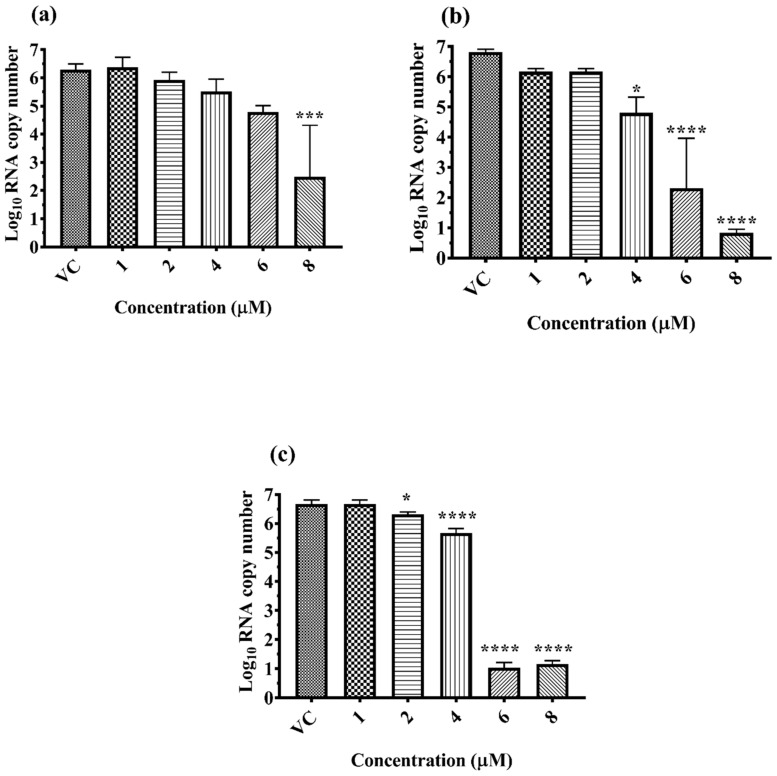
Effect of α-Mangostin on DENV-2 RNA levels, quantified by qRT-PCR under pre-treatment (**a**), co-treatment (**b**) and post-treatment (**c**) conditions. The fold change was compared with the virus control (VC) and presented logarithmically. All the values are expressed as mean ± SD of three experiments **** *p* < 0.0001; *** *p* = 0.0002; and * *p* = 0.0194 vs. control.

α-Mangostin treatment resulted in a significant reduction in the log10 titer of viral RNA copy number under pre-, co- and post-treatment conditions (Figure 2a–c). A significant 3 log10 titer (*p* = 0.004) decrease in copy number of DENV-2 RNA was observed for pre-treatment at 8 µM concentration (Figure 2a). A ~2 log10 (*p*= 0.0226), ~4.5 log10 (*p* < 0.0001) and 6 log10 (*p* < 0.0001) reduction of viral RNA titer was observed at 4, 6 and 8 µM concentration of α-Mangostin under co-treatment conditions (Figure 2b). A significant 1 log10, 5.6 log10 and 5.5 log10 (*p* < 0.0001 for all) reduction of viral RNA was observed at 4, 6 and 8 µM concentrations of α-Mangostin when it was added 24 h after infection (Figure 2c).

**Figure 3:**
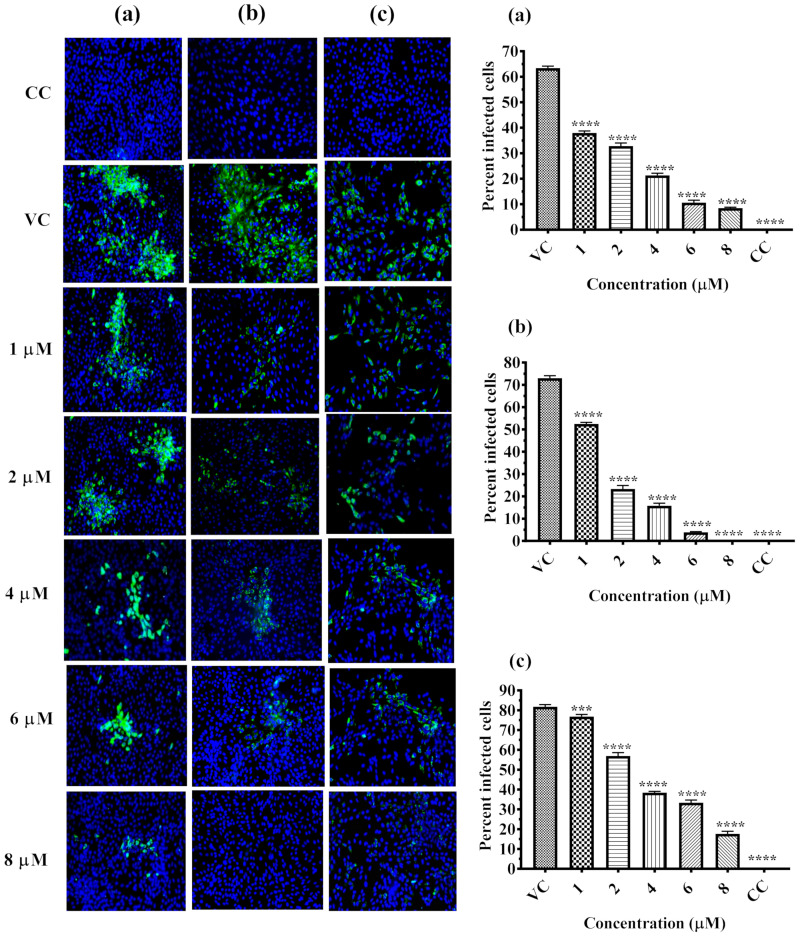
Microscopic images of Immunofluorescence assay. Immunoflourescent images of DENV-2 infected Vero ccl81 cell lines under pre-treatment, co-treatment and post-treatment conditions. Virus infected cells appear green in color Percentage of infected Vero ccl81 cell line in cultures infected with virus with different concentrations of compound under pre-treatment (**a**), co-treatment a (**b**) and post-treatment (**c**) conditions. All the values are expressed as mean ± SD of three experiments ****, *p* < 0.0001 and ***, *p* = 0.0002 vs. control.

IFA was performed to detect the DENV-2 antigen in the tested cells as an indicator of infection and to determine the extent of inhibition by α-Mangostin at different concentrations. As shown in Figure 3a, there was a strong inhibition of the viral antigen and dose-dependent reduction in the percent of infected cells pre-treated with α-Mangostin. A similar reduction pattern of infection was also observed in both co- and post-treated cells in comparison with the VC (Figure 3b,c). Interestingly, 100% reduction of virus infection was observed in cells during co-treatment with 8 µM of α-Mangostin (Figure 3b).

### 2.3. In Silico Interaction Studies of α-Mangostin with DENV Protein Targets

In order to investigate the possible mechanism of action of α-Mangostin as an anti-DENV compound, computational docking studies were carried out with the widely known DENV drug targets using AutoDock Vina. After docking, the best pose based on its conformation and docking energy was selected. Binding affinity calculations were carried out using the scoring function algorithm implemented in AutoDock Vina.

**Figure 4:**
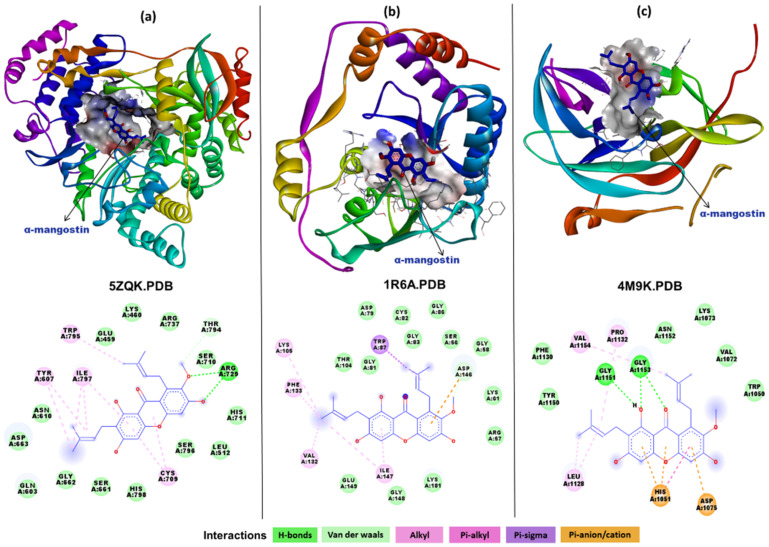

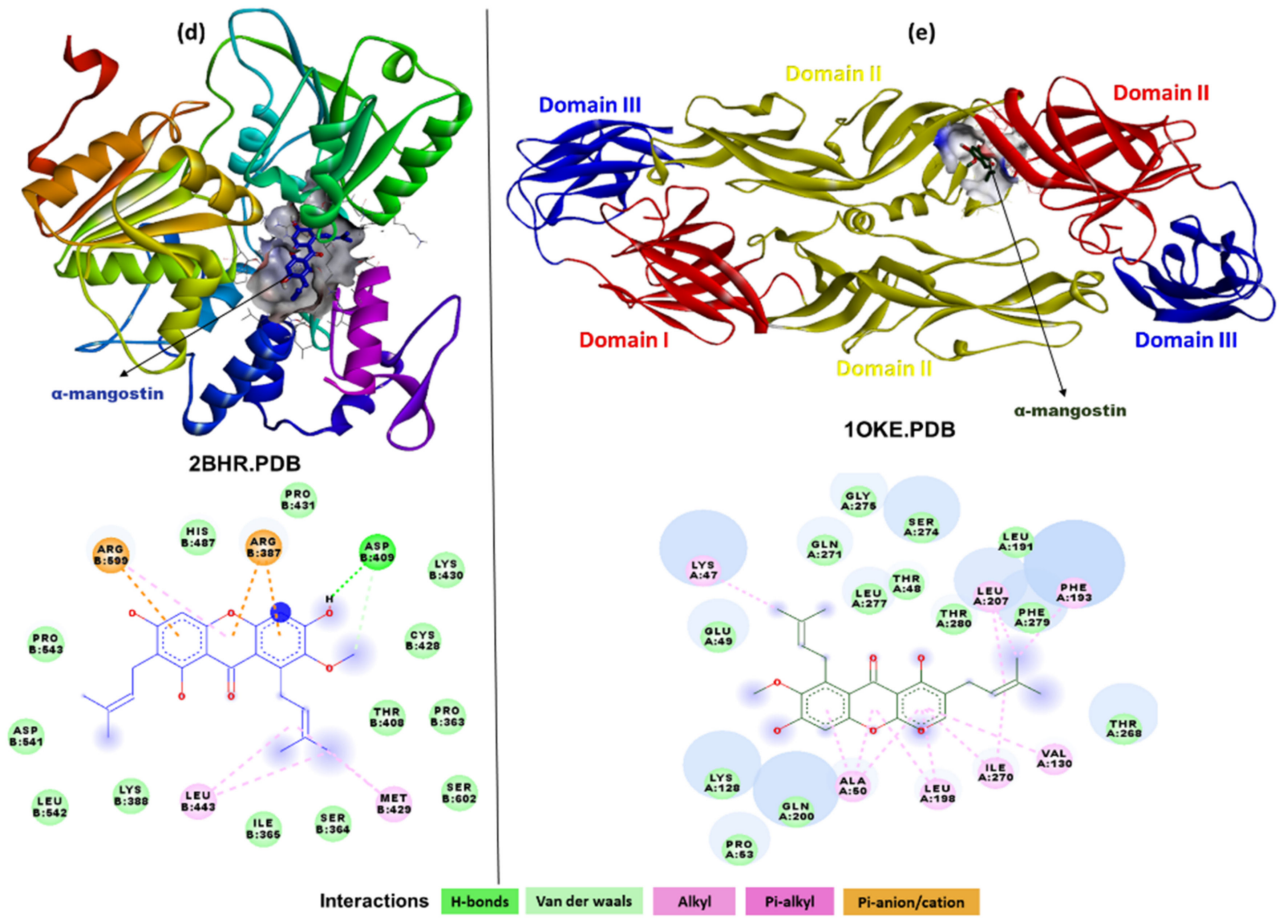
Molecular interactions of α-Mangostin with DENV non-structural and structural protein targets. Ribbon diagram with the solvent surface rendered view (probe radius 1.6 Å) and 2-dimensional interaction diagram showing α-Mangostin (in blue stick model) interaction with DENV (**a**) NS5 RdRP domain (**b**) NS5 methyltransferase domain (**c**) NS2-NS3 protease domain (**d**) NS3 helicase domain. (**e**) Envelope glycoprotein complex. The different types of interactions are represented by different colors mentioned in the interactions color panel. The interactions were visualized and analyzed using Biovia Discovery studio client 2017.

The docking interaction analysis of α-Mangostin with DENV NS5 RdRp domain (Figure 4a) revealed that the compound docked with strong binding affinity (−8.2 kcal/mol) and binds to a potential binding site near the catalytic site interacting with the residues of all three conserved motifs (Q598-N614, G662-D664 and C709-R729) as well as residues of the priming loop (H786-M809). The interaction analysis showed that there were two hydrogens bonds formed with Arg729 (belonging to motif II) and the hydroxyl group of α-Mangostin. There were multiple hydrophobic interactions including alkyl and pi-alkyl bonds noted with residue Tyr607 (motif I), Cys709 (motif II), and Trp795, Ile797 (priming loop).

The docking interaction analysis of α-Mangostin with DENV NS5 methyltransferase domain showed good binding affinity (−7.9 kcal/mol) and binding at a site between the catalytic site (Lys61, Asp146, Lys181, Glu218) and the proposed SAH binding site (Lys105, Thr104, Gly83, Cys82, Gly81, Val132, Phe133 and Ile147) (Figure 4b). A single electrostatic interaction was observed with the catalytic residue Asp146, whereas other catalytic residues—Lys61 and Lys181—formed Van der Waals interactions. There were several hydrophobic interactions noted forming alkyl and pi alkyl interactions with Lys105, Val132, Phe133 and Ile147. Trp87 interacted with Pi-sigma bonding.

In order to evaluate the binding ability of α-Mangostin to the complete NS5 protein inclusive of the methyltransferase and RdRp domains, docking interaction analyses were also carried out with the structure of the complex. The results revealed that majority of the docking poses showed interactions with the methyltransferase domain with the highest binding affinity of −7.7 kcal/mol. Some of the poses also showed an interaction with the RdRp domain with highest binding affinity of −7.8 kcal/mol. Interacting residues (Figure S1) were equivalent to those observed in the individual domain-based docking study (Figure 4a,b). The difference in binding affinities in the two cases of the individual domain-based docking versus the complete complex may be attributed to the difference in solvent accessibility as an effect of the conformation search space defined while docking (Figure S1).

The DENV NS2B-NS3 protease is one of the most attractive targets for anti-DENV inhibitor compounds. Other than the main catalytic site (His51, Asp75, Ser135), a specific allosteric binding site (Trp89, Thr120, Gly121, Glu122, Ile123, Gly124, Gly164, Ile165, Ala166, and Gln167 on one side and Lys73, Lys74, Asn152, Val78, Gly82, and Met84 on the other side, the last three of which are from the NS2B) has also been proposed previously for non-competitive and non-peptidic inhibitors. α-Mangostin docking with DENV protease (Figure 4c) showed that the best docked pose of the compound (binding affinity −7.1 kcal/mol) interacted with residues of both the catalytic and allosteric site. Pi-anion and pi-alkyl interactions were formed between the phenol rings of α-Mangostin and the catalytic residue His51 (1051 in Figure 4c). Another catalytic residue Asp75 (1075 in Figure 4c) interacted with a Pi-cation interaction. There were three hydrogen bonds formed with the allosteric site residue Gly151 (1151 in Figure 4c), Gly1153 (1153 in Figure 4c). Asn152, Lys73 and other vicinity residues in the allosteric site were noted to have interacted with Van der Waals and hydrophobic interactions.

The docking interaction of α-Mangostin with NS3 helicase domain (Figure 4d) showed that the binding affinity for the best pose was −8.2 kcal/mol. There were pi-cation interactions formed between residue Arg387 and Arg599 and the phenol rings of the compound. Asp409 was the only residue which was found to interact with hydrogen bonding. Other residues in the vicinity showed Van der Waals and hydrophobic interactions. Though the binding affinity score was high, the interacting residues did not belong to any of the crucial functional sites such as the NTP binding site or the known conserved motifs in the NS3 helicase domain.

The docking interaction analysis of α-Mangostin with the E glycoprotein (Figure 4e) revealed that the compound binds to the hydrophobic pocket accommodating the residues of the fusion peptide (268–280). The binding affinity was observed as −7.0 kcal/mol with multiple hydrophobic interactions but no hydrogen bond formed between the compound and the protein complex. The residues Lys47, Ala50, Val130, Phe193, Leu198, Leu207 and Ile270 interacted with alkyl and pi-alkyl interactions.

## 3. Discussion

The development of new therapeutics against DENV is necessary to reduce the burden of dengue. The disease severity of DENV-2 in terms of severe dengue is significantly much higher than DENV-1, 3 and 4 [19]. Biodiversity of natural products serves as an excellent source for discovery of novel antivirals with new structure–activity relationships and developing effective prophylactic/therapeutic strategies against viral infections. A variety of natural products such as polysaccharides, flavonoids, alkaloids, terpenoids, polycyclic quinones and phenolics have been identified as potent antiviral sources against DENV through in vitro cell-based approaches [18,20,21,22,23,24]. In vitro assays depend on the virus’ ability to infect and replicate in specific cell lines as the cell culture system provides a rapid and reliable method to grow viruses at higher titers [25]. In the present study, among the six compounds that showed antiviral activity against DENV-2, α-Mangostin was the only compound that showed maximum inhibition of DENV-2 under all the treatment conditions (pre-, co- and post-treatment). This compound is generally purified from the pericarp of the Mangosteen fruit (*Garcinia mangostana* Linn.) in some Southeast Asian nations [26]. Several studies reported that it possesses a wide range of biological activities including anti-inflammatory, anti-allergic, antiviral, antibacterial, antifungal, anti-parasitic, antioxidant, and anti-cancer properties [27,28]. Earlier studies have also shown that α-Mangostin can inhibit DENV in peripheral blood mononuclear cells and hepatic cell lines and down-regulate the expression of pro-inflammatory cytokines [29,30]. These studies have shown the effect of α-Mangostin post-infection. The present study has utilized α-Mangostin purified from a natural source and shown the antiviral activity with lower concentrations under pre-, co- and post-treatment conditions in Vero CCL81 cell line, which is a highly permissive cell line. Moreover, in the present study, docking analyses involving α-Mangostin and various DENV non-structural and structural proteins have been performed to find the possible mechanisms of DENV inhibition by α-Mangostin.

The in vitro findings that α-Mangostin can inhibit DENV under co-treatment condition indicate virucidal activity. This inhibition under the co-treatment condition could be due to the binding of α-Mangostin to the virus surface glycoprotein E, which was also reflected in the in silico analysis. Docking studies revealed that α-Mangostin can interact with the E protein and binds specifically in the hydrophobic pocket in which residues of the fusion peptide are accommodated [31]. On the other hand, DENV inhibition on the pre-treatment of cells raises the possibility of α-Mangostin, possibly interacting with a cellular receptor leading to a reduction of DENV uptake. In Vero cells, heparin sulfate, and a 74-kDa protein has been reported to act as a receptor for DENV binding [32]. It is possible that α-Mangostin can interact with this receptor or other unidentified receptors to inhibit the DENV entry.

The in vitro inhibition of DENV-2 by α-Mangostin post-infection proposes that α-Mangostin can interfere with the functioning of multiple non-structural proteins including the NS5 methyltransferase, NS5 RdRp and NS2B-NS3 protease and may reduce the replication of DENV-2. These findings from in vitro experiments enhanced our understanding on the compound’s efficacy during the later stages of the virus life cycle during the replication phase. DENV NS5 protein is a potential target for the development of anti-dengue agents since it possesses both the RdRp domain essential for viral RNA synthesis and the methyl transferase domain involved in 5′ RNA capping and methylation to promote efficient RdRp activity [33]. The RdRp domain consists of three subdomains: thumb, fingers, and palm. The thumb subdomain contains the priming loop, which extends into a double-stranded RNA binding site and is hypothesized to undergo a conformational change during the de novo initiation of RNA synthesis [34]. In the molecular docking analysis, α-Mangostin interacted at the RNA binding site occupying the residues of three conserved motifs, motif B (Q598-N614), motif C (G662-D664) and motif E (C709-R729), as well as residues of the priming loop (H786-M809) [35,36,37]. R729 is highly conserved among flaviviruses and is involved in coordinating the phosphate groups. The interaction of α-Mangostin with R729 might impact the de novo synthesis and elongation of RNA [38]. Investigations of docking with the NS5 methyl transferase domain revealed the interaction of α-Mangostin with residues in the catalytic domain, suggesting that it might inhibit methyl transferase activity. Furthermore, based on the in silico results of docking with the complete NS5, among the two domains of NS5, preferential binding was noted for the methyltransferase domain. The α-Mangostin interaction with DENV NS2B-NS3 protease revealed that the compound bound between the main catalytic site and previously proposed allosteric binding site for non-competitive and non-peptidic inhibitors [39]. Though α-Mangostin interacted with both catalytic and allosteric site residues, it lacks the interaction with Ser135, which plays a key role in the protease enzymatic activity.

The possible anti-DENV mechanism of α-Mangostin was also investigated by docking it with the DENV structural envelope glycoprotein complex. α-Mangostin bound at the furin cleavage site by forming weaker hydrophobic interactions. This was in agreement with the findings where inhibition of DENV on α-Mangostin co-treatment was observed.

Overall, the present study concluded that α-Mangostin can inhibit the DENV-2 under the three testing conditions of pre-, co- and post-treatment. Data from the above-mentioned molecular docking experiments indicated that α-Mangostin can interact with multiple DENV protein targets, thus suggesting the compound’s ability to suppress DENV production at different stages of its replication cycle. All the in silico interaction studies need validation by experimental studies. Apart from direct interaction with DENV, α-Mangostin might also exert indirect effects on the cells to inhibit the virus replication. α-Mangostin is a potent modulator of immune response. It is known to activate the human stimulator of the interferon (IFN) gene (STING) protein and can induce the expression of IFN-stimulated genes (ISG) in an IFN-independent manner, which is mediated through IFN regulatory factor (IRF)-3 [40,41,42]. Thus, in Vero cells that are deficient in producing IFN, α-Mangostin might cause the modulation of an innate immune response and contribute to the inhibition of DENV. In the present study, though the antiviral activity of α-Mangostin was assessed against only DENV-2, the compound needs to be tested against the other serotypes as well. Our earlier study had shown that α-Mangostin inhibits chikungunya virus (CHIKV) in infected C57BL/6 mice [43]. Since CHIKV and DENV infection results in similar types of clinical symptoms, α-Mangostin might be a promising candidate for treatment of dengue-like illness. However, more evidence from studies in animal models followed by clinical trials are needed to conclude this. The present study confirms the anti-DENV-2 activity of α-Mangostin in cell lines and needs to be supported by in vivo studies.

## 4. Materials and Methods

### 4.1. Cells and Virus Maintenance

Vero ccl81 cell line (ATCC^®^ CCL 81™), derived from kidney of African green monkey was used for this work. The cells were grown at 37 °C in 5% CO_2_ and maintained in Minimal Essential Medium (MEM) with 10% Fetal bovine serum albumin (FBS, Gibco, Technologies, New York, NY, USA) and 200 µg/mL antimycotic antibiotic solution (Sigma Aldrich, St Louis, MO, USA). DENV-2 (Strain no. 803347) stock was prepared using in C6/36, a mosquito cell line. Throughout this study, 0.1 Multiplicity of Infection (MOI) of virus stock was used for infection.

### 4.2. Compounds Stock Preparation

A total of 47 synthesized natural compounds obtained from CSIR-IICT, Hyderabad were used for screening. Stock solutions (10mM) of compounds were prepared by diluting compounds in DMSO (0.1%) and purified by filtering through syringe filter of 0.2 µM pore size. The stock solutions of the compounds were preserved at −20 °C for further work.

### 4.3. Cell Cytotoxicity Assay of Compounds

The cytotoxicity activity of 47 compounds was evaluated by 3- (4,5-dimethythiazol-2-yl)-2,5-diphenyl tetrazolium bromide (MTT) assay as described previously [44]. A confluent monolayer of Vero ccl81 cells was prepared in 96-microtiter-well plate at a density of 20,000 cells per well. Different concentrations of respective compounds were prepared by using stock solution and diluted in minimum essential medium. The concentrations used for cell cytotoxicity studies were 100 µM, 50 µM, 25 µM, 12.5 µM 6.25 µM, 3.125 µM and 1.56 µM. Next the cells were treated with the different compounds and kept for 5 days incubation at 37 °C with 5% CO_2_. After incubation, 10 µL of MTT solution was added to the cells and incubated for 3 h at 37 °C. The cells were then treated with 100 µL of isopropyl alcohol (5% 0.1 N HCl in Isopropanol) and incubated for one hr. at 37 °C. The readings were taken in a microplate reader (infinite F50, Tecan, Switzerland) at a wavelength of 570 nm with reference filter at 690 nm. Percentage cytotoxicity or viability was calculated in comparison with cells untreated with compounds.

### 4.4. Antiviral Assays

The antiviral effect of the compounds (at the maximum non-toxic dose) were assessed before infection (pre-treatment of cells), during infection (co-treatment) and after infection (post-treatment). During pre-treatment, the cells (50,000 cells/well) were pre-treated with compound at 37 °C for 24 h. Then the compound containing culture supernatant was removed and the cells were infected with 0.1 MOI of DENV-2. After infection, the cells were washed twice to remove the unbounded virus and kept for incubation by adding maintenance media (MEM with antibiotics and 2% Fetal bovine serum). In co-treatment, the virus was mixed with different concentrations of the compound and the mixture was used for infecting cells for duration of one h. For post-treatment, the cells were infected with 0.1 MOI DENV-2 for 1 h. and treated with the compound after 24 h. For all types of treatments, the cells were incubated for five days post-infection. In the virus control, wells contain only infected cells without any treatment while the cell control contains uninfected cells only. In all conditions, plates were frozen at −80 °C and thawed to collect cell supernatant for quantitative estimation of viral RNA.

For the compound α-Mangostin which showed antiviral activity, the assays were repeated under pre-, co- and post-treatment conditions with different concentrations of the compounds as described earlier. The collected supernatant was used for determination of viral genomic RNA level by real-time RT-PCR and titer of infectious virus particle by focus forming unit assay. All the experiments were performed in triplicates in two independent trials.

#### 4.4.1. Quantitative Reverse Transcription Polymerase Chain Reaction (qRT-PCR) for Estimating Viral RNA Copy Number

qRT-PCR was performed to evaluate the effects of α-Mangostin on DENV-2 replication by quantifying the DENV-2 RNA copy number based on a method described previously [35]. Briefly, A QIAamp Viral RNA mini kit (Qiagen, Hilden, Germany) was used for extraction of viral RNA following manufacturer’s instruction. One step qRT-PCR using a commercial kit (Invitrogen SuperScript III Platinum One-step qRT-PCR Kit) was used for detection and quantitative estimation of RNA. Oligonucleotides sequences were used as described earlier [45]. The amplification conditions include incubation at 50 °C for 30 min followed by 95 °C for 10 min, and 40 cycles of 95 °C for 15 s with 60 °C for 1 min. The copy numbers of the samples were calculated based on standard graph generated using Ct values of tenfold dilutions of in vitro transcribed viral RNA with known copy number. The assays were repeated for the compounds which showed inhibitory activity compared to the virus control.

#### 4.4.2. Focus Forming Unit (FFU) Assay

FFU assay was used for quantification or determining the infectious virus particle. The assay was performed as described earlier [46]. Approximately, 20,000 Vero ccl81 cells/well were seeded in a 96 well plate and incubated for 24 h to form a confluent monolayer. Ten-fold serial dilution of the culture supernatant was done with fresh MEM and 100 µL was transferred to the monolayer of cell and the plates were incubated for 1 h. After incubation, MEM with 2% FBS and 1.8% carboxy methyl cellulose (overlay media) were added and incubated at 37 °C for 5 days in a CO_2_ incubator. After incubation, PBST was used for washing and fixation of cells was done by chilled acetone with methanol (1:1 ratio). Then, blocking buffer (1% bovine serum albumin dissolved in PBS buffer) was added to each well and incubated for 40 min at 37 °C and cells were washed two times with PBST. Cells were incubated at 37 °C for 40 min with primary antibody (anti-prM–dengue antibody with dilution 1:200) followed by addition of secondary antibody (anti-mouse IgG HRP conjugate with dilution 1:1000) and incubation for 40 min. Cells were washed twice with PBST before and after secondary treatment. Blue color foci were developed by incubating the cells with True Blue Peroxidase Substrate (KPL) in dark at room temperature for 15 min. Substrate was removed after blue tinge formation and dried before counting the foci. Virus titer was determined by counting number of foci after scanning in scanner.

#### 4.4.3. Immunofluorescence Assay

IFA assay was performed for the quantitative estimation of virus infectivity. The similar protocol was carried out with minor modifications described earlier [34]. To measure the dose dependent infectivity, only co-treatment was performed by incubating cells with different concentrations of compound with 0.1 MOI virus. Approximately 50,000 Vero ccl81 cells per well were seeded in a 24-well plate (Tissue Culture Test Plate 24, TPP, Switzerland) with a coverslip placed in each well. The cells were allowed to form a confluent monolayer in a 5% CO_2_ incubator at 37 °C. The cells adhered to the cover slips were fixed by chilled Acetone and Methanol in 1:1 ratio for 15 to 20 min and blocked with 1% bovine serum albumin (BSA) (Sigma-Aldrich St. Louis, MO, USA) followed by PBST wash. The cells were incubated with anti-PRM dengue antibody (1:50) followed by incubation with anti-mouse IgG secondary antibody (1:50 dilution) for 1 h. After incubation, the cover slips were mounted onto slides with a drop of mowiol (mounting solution) containing 4′,6-diamidino-2-phenylindole, dihydrochloride (DAPI) (nuclear stain) (Sigma-Aldrich St. Louis, MO, USA). The slides were visualized under a fluorescent microscope (20× magnification) (EVOS Floid cell imaging station, Thermo Fisher Scientific, Bedford, MA, USA) and further analyzed using ImageJ software. For each coverslip, at least four to five fields were counted and the average was considered for analysis.

### 4.5. Compound, Molecular Modelling and Docking Studies with Viral Proteins Targets

The two-dimensional structure of α-Mangostin was generated using ChemDraw^®^ Professional 16.0.1.4 software. All available three-dimensional (3D) crystallographic structures of the DENV-2 target proteins in the Protein Data Bank (PDB) including DENV-2 envelope glycoprotein (1OKE.pdb), DENV-2 non-structural proteins including NS2B-NS3 protease (4M9K.pdb), NS3 helicase domain (2BHR.pdb), NS5 methyltransferase domain (1R6A.pdb) and NS5 RdRP (RNA dependent RNA Polymerase) domain structure (5ZQK.pdb) were retrieved from the Protein Data Bank (http://www.rcsb.org, accessed on 2 May 2020). The structure of the complete NS5 complex inclusive of the methyl transferase domain with the RdRp domain in 5ZQK.pdb was also for a separate protein docking study [47]. However, based on the role of the NS2B as a co-factor for the NS3-based protease activity, we used the NS2B-NS3 complex rather than the complex of NS3 protease with the NS3 helicase (2VBC.pdb).

The ligand-protein docking interactions of all the DENV target crystal structures were simulated using AutoDock Vina. The target structures were pre-processed and minimized by adding polar hydrogens and gasteiger charges using Autodocktools (ADT). The grid box parameters were set such that the search is performed over the entire protein surface. Default values were used for all the other docking parameters. The ligand for the docking studies was also pre-processed by AutodockTool (ADT). In case of the NSP5 methyltransferase domain, the coordinates of RTP (ribavirin tri-phosphate), the co-crystalized inhibitor, were deleted during the docking study. The binding site predictions prior to docking studies, the interaction analysis and molecular visualization of docked complexes were performed using BIOVIA Discovery Studio 2020 client software package.

## Data Availability

Data sharing is not applicable to this article.

## Supplementary Materials

The following are available online. Figure S1: Molecular interactions of α-Mangostin with DENV non-structural protein 5 (NS5) complex of methyltransferase (MTase) and RdRp (a) NS5 methyltransferase domain docking pose (b) NS5 RdRp domain docking pose.

## Sample Availability

Samples of the compounds are not available from the authors.
