# Supplementary material for: In Vitro Antiviral Activity of α-Mangostin against Dengue Virus Serotype-2 (DENV-2)
Source: Molecules. 2021 May 19;26(10):3016. doi: 10.3390/molecules26103016 (PMC8158742; doi:10.3390/molecules26103016)
Supplement: Supplementary file 1 [file molecules-26-03016-s001.zip › molecules-1164394-supplementary.pdf]

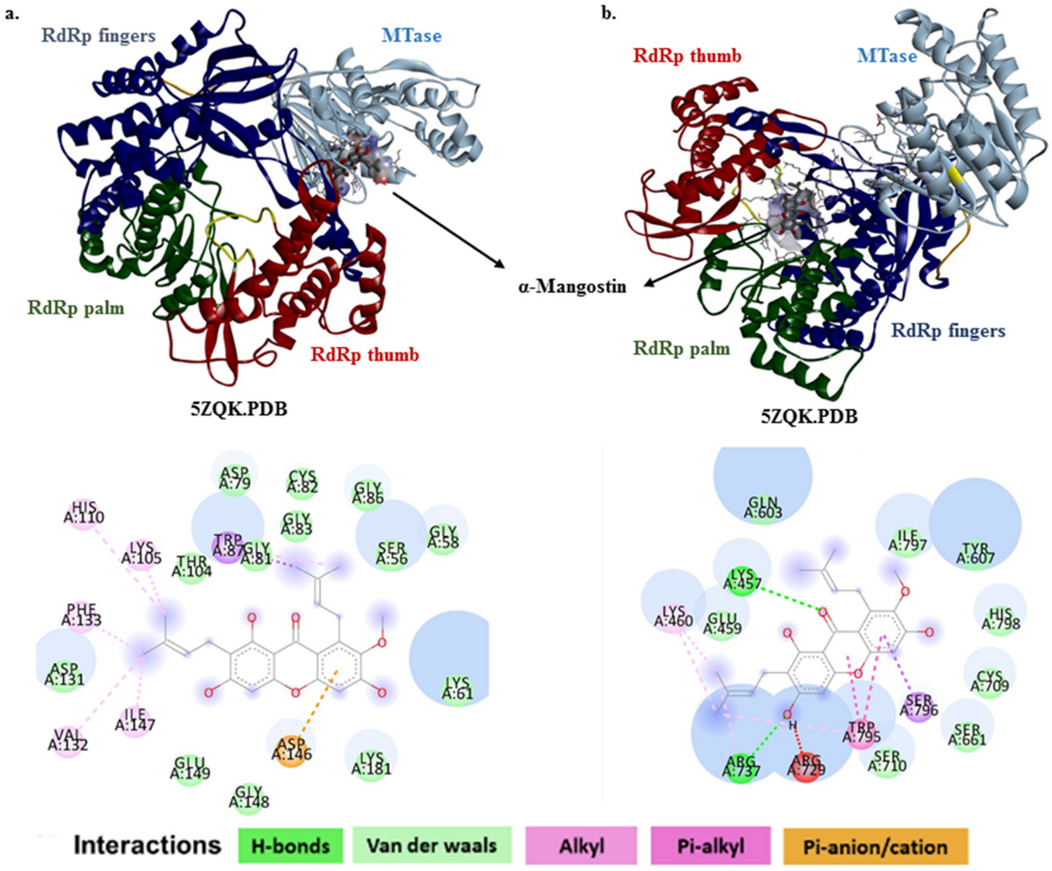

Figure S1. Molecular interactions of α-Mangostin with DENV non-structural protein 5 (NS5) complex of methyltransferase (MTase) and RdRp (**a**) NS5 methyltransferase domain docking pose (**b**) NS5 RdRp domain docking pose.
